# *Coxiella burnetii* and *Leishmania mexicana* residing within similar parasitophorous vacuoles elicit disparate host responses

**DOI:** 10.3389/fmicb.2015.00794

**Published:** 2015-08-07

**Authors:** Jess A. Millar, Raquel Valdés, Fenil R. Kacharia, Scott M. Landfear, Eric D. Cambronne, Rahul Raghavan

**Affiliations:** ^1^Department of Biology and Center for Life in Extreme Environments, Portland State University, Portland, ORUSA; ^2^Department of Molecular Microbiology and Immunology, Oregon Health and Science University, Portland, ORUSA

**Keywords:** *Coxiella burnetii*, *Leishmania mexicana*, parasitophorous vacuole, isoform, miRNA

## Abstract

*Coxiella burnetii* is a bacterium that thrives in an acidic parasitophorous vacuole (PV) derived from lysosomes. *Leishmania mexicana*, a eukaryote, has also independently evolved to live in a morphologically similar PV. As *Coxiella* and *Leishmania* are highly divergent organisms that cause different diseases, we reasoned that their respective infections would likely elicit distinct host responses despite producing phenotypically similar parasite-containing vacuoles. The objective of this study was to investigate, at the molecular level, the macrophage response to each pathogen. Infection of THP-1 (human monocyte/macrophage) cells with *Coxiella* and *Leishmania* elicited disparate host responses. At 5 days post-infection, when compared to uninfected cells, 1057 genes were differentially expressed (746 genes up-regulated and 311 genes down-regulated) in *C. burnetii* infected cells, whereas 698 genes (534 genes up-regulated and 164 genes down-regulated) were differentially expressed in *L. mexicana* infected cells. Interestingly, of the 1755 differentially expressed genes identified in this study, only 126 genes (~7%) are common to both infections. We also discovered that 1090 genes produced mRNA isoforms at significantly different levels under the two infection conditions, suggesting that alternate proteins encoded by the same gene might have important roles in host response to each infection. Additionally, we detected 257 micro RNAs (miRNAs) that were expressed in THP-1 cells, and identified miRNAs that were specifically expressed during *Coxiella* or *Leishmania* infections. Collectively, this study identified host mRNAs and miRNAs that were influenced by *Coxiella* and/or *Leishmania* infections, and our data indicate that although their PVs are morphologically similar, *Coxiella* and *Leishmania* have evolved different strategies that perturb distinct host processes to create and thrive within their respective intracellular niches.

## Introduction

Macrophages that phagocytize pathogens and recruit other immune cells are critical for the elimination of potential infections. Within macrophages, engulfed pathogens are transported inside phagosomes that later fuse with lysosomes to generate the phagolysosome. Most pathogens are degraded within the phagolysosome, which has a very harsh environment (low pH, high concentration of lysosomal hydrolases, presence of cationic peptides etc.; Kinchen and Ravichandran, 2008; Flannagan et al., 2009). Several pathogens have evolved strategies to survive and replicate within macrophages: *Toxoplasma gondii* prevents the fusion of its vacuoles with the endosomal pathway; *Salmonella enterica* Typhimurium, *Mycobacterium tuberculosis*, and *Legionella pneumophila* block maturation of phagosomes into phagolysosomes; *Shigella flexneri* and *Listeria monocytogenes* escape into cytoplasm from phagosomes before lysosomal fusion; *Trypanosoma cruzi* escapes from phagosomes after fusion with lysosomes (Swanson and Fernandez-Moreia, 2002; Flannagan et al., 2009).

Unlike most other pathogens, *Coxiella* (a bacterium) and *Leishmania* (an eukaryote) have independently evolved the ability to thrive in a parasitophorous vacuole (PV) that is derived from the fusion of phagosomes with lysosomes (Voth and Heinzen, 2007; Alix et al., 2011). *Coxiella burnetii* (the only defined species within this genus) causes human Q fever and chronic endocarditis. The bacterium is shed in milk, urine, and birth products of animals, and can survive in the environment via a “spore-like” form called the small cell variant (SCV). *C. burnetii* is usually acquired via inhalation, and initially infects alveolar macrophages but then spreads to mononuclear phagocytes of other tissues. Within the macrophage, SCV transforms into a metabolically active form called the large cell variant (LCV), and multiple *Coxiella*-containing vacuoles merge to form a single large vacuole that fuses with endolysosomal vesicles to give rise to the mature *Coxiella* PV (van Schaik et al., 2013).

*Leishmania* is a genus of trypanosomatid parasite that comprises several species of medical and veterinary importance that cause cutaneous, mucocutenous, or visceral diseases. It has a dimorphic lifecycle that alternates between an extracellular promastigote form in insect vectors and an intracellular amastigote from in mammalian hosts (Herwaldt, 1999). The primary host cells of *Leishmania* are macrophages, but it can also infect neutrophils, fibroblasts, and dendritic cells (Contreras et al., 2014). Similar to the biogenesis of *Coxiella* PV, the *Leishmania*-containing vacuole also fuses with endolysosomal vesicles to give rise to the mature *Leishmania* PV. However, the morphology of PV varies among different *Leishmania* species. In several species, including *L. donovani*, *L. infantum*, and *L. major*, only one or two amastigotes reside within each PV, which segregates into new vacuoles after parasite replication. In contrast, as observed for *Coxiella* PVs, parasites of the *Leishmania mexicana* complex such as *L. mexicana* and *L. amazonensis* form communal PVs that continuously enlarge as the parasites replicate (Real et al., 2010). Interestingly, coinfection studies have shown that PVs formed by *L. amazonensis* amastigotes can fuse with *C. burnetii* PVs but not with PVs containing *L. major* amastigotes, suggesting that the intracellular niches generated by *L. mexicana* complex parasites and *Coxiella* may be compositionally rather similar (Veras et al., 1995; Rabinovitch and Veras, 1996; Real et al., 2010; Beare et al., 2011; Newton and Roy, 2011).

Both *Coxiella* and *Leishmania* actively participate in the creation of their respective PVs, which are intracellular compartments distinct from canonical phagolysosomes. To begin to understand how the two distantly related pathogens generate phenotypically similar PVs, we compared host gene expression in human macrophage cells (THP-1) infected with either *C. burnetii* or *L. mexicana*. Our data show that the bacterium and the eukaryote elicit distinct host messenger RNA (mRNA) and microRNA (miRNA) responses, indicating that despite their superficial similarity, generation, and maintenance of the *Coxiella* PV and *Leishmania* PV involve distinct host processes.

## Materials and Methods

### *C. burnetii* and *L. mexicana* Infection of THP-1 cells, RNA Extraction, and RNA-seq

THP-1 cells (TIB-202; ATCC) were maintained in RPMI 1640 medium (Gibco) supplemented with 10% fetal calf serum (Gibco) at 37°C in 5% CO_2_. Cells were incubated in the presence of 200 nM phorbol 12-myristate 13-acetate (PMA; EMD Biosciences) for 24 h to induce differentiation into adherent, macrophage-like cells. Prior to infection, PMA-containing medium was replaced with fresh RPMI without PMA. Cells were infected with either *C. burnetii* (Nine Mile phase II, RSA 493) or promastigotes of *L. mexicana* (MNYZ/BZ/62/M379) at an approximate multiplicity of infection of 25 and incubated for 5 days. Growth medium was replaced every two days and formation of *Coxiella* and *Leishmania* PVs was monitored microscopically. At 5 days post-infection, growth medium was replaced with 1 ml of TRI reagent (Life Technologies) and total RNA was extracted, and genomic DNA was removed by DNase (Life Technologies) treatment, as per instructions. RNA from two samples each of uninfected, *Coxiella*-infected, and *Leishmania*-infected THP-1 cells were used to prepare mRNA and small RNA Illumina sequencing libraries. To analyze gene expression, the six mRNA libraries were pooled into a single lane of an Illumina HiSeq 2000 (2 × 75 cycles). For miRNA identification, the six small RNA libraries were pooled into a single Illumina Miseq lane (1 × 50 cycles). All RNA-seq reads are available at National Center for Biotechnology Information Sequence Read Archive (Accession SRP045986).

### Mapping Sequencing Reads and Identification of Differentially Expressed Genes

Reads were cleaned by removing adapters and were filtered by quality (>Q20) and length (>50 bp) using Trimmomatic v0.30 (Bolger et al., 2014). *Homo sapiens* reads were filtered for possible contamination by mapping to *C. burnetii* genome (NC_002971.3) using BWA MEM v0.7.5 (Li and Durbin, 2010) and *L. mexicana* genome (NZ_CADB00000000.1) using Tophat v2.0.11 (Kim et al., 2013). Final clean reads were mapped to *H. sapiens* Genome Reference Consortium Human Build 37 (GCF_000001405.13) using CLC Genomic Workbench v6.5. To identify differential gene expression, replicate data were pooled for pairwise comparisons and quantile normalized using CLC Genomic Workbench v6.5. Genes were filtered based on at least 10 raw reads mapping to each sample, and a log2 transformed fold change of one SD above or below the mean. Differentially expressed genes were chosen based on significance (*P* < 0.05, FDR-corrected beta-binomial distribution test). Raw read counts mapped to each mRNA isoform were exported from CLC into EBSeq (Leng et al., 2013) and differential expression of isoforms was determined based on significant EBSeq values (*P* < 0.05, FDR-corrected).

For quantitative PCR (qPCR) validation of gene expression, 1 μg of DNase-treated RNA, and oligo-dT primers were used to prepare cDNA (Thermo Scientific). A subset of genes involved in host cell death (TGFB2, RIPK2, CYR61, CYP1B1, NFKBIA) was selected and qPCR was performed using SYBR green on an Agilent Mx3000P System. Fold difference value for each gene was calculated using the 2^-ΔΔCT^ method with GAPDH as the control. As shown previously (Raghavan et al., 2012), to assess the correlation between expression estimates from RNA-seq and qPCR, we calculated the Pearson correlation coefficient between fold difference values calculated by each method for *Coxiella* – and *Leishmania*-infected cells.

### Gene Ontology (GO) Analysis and Protein-Protein Interaction Networks

GO terms were found using Database for Annotation, Visualization and Integrated Discovery (DAVID), and the GO FAT filter. GO-term enrichment tests were also performed with DAVID (Huang et al., 2009a,b). Kyoto Encyclopedia of Genes and Genomes (KEGG) pathways over-represented among differentially expressed genes were chosen based on the level of statistical significance (*P* < 0.01). Protein–protein interaction networks were visualized using STRING 9.1 (Franceschini et al., 2013). Proteins unconnected to the main graph were removed. Markov Clustering was performed on STRING confidence scores using an inflation factor of two to visualize subgraphs of interacting protein processes (Brohée and van Helden, 2006). GO-terms were overlaid onto the graphs using STRING to identify what processes were represented in the separate subgraphs.

### Identification of miRNAs

Sequencing reads were cleaned by removing adapters and filtered by quality (>Q20) and length (>15 bp) using Trimmomatic (Bolger et al., 2014). Replicate data was pooled and miRNAs were identified using CLC based on having an average of at least 10 reads mapped to mature 5′ or 3′ miRNAs annotated in mirBase (Kozomara and Griffiths-Jones, 2014).

## Results and Discussion

### *C. burnetii* and *L. mexicana* Infections Induce Robust but Non-Overlapping Host Responses

**FIGURE 1:**
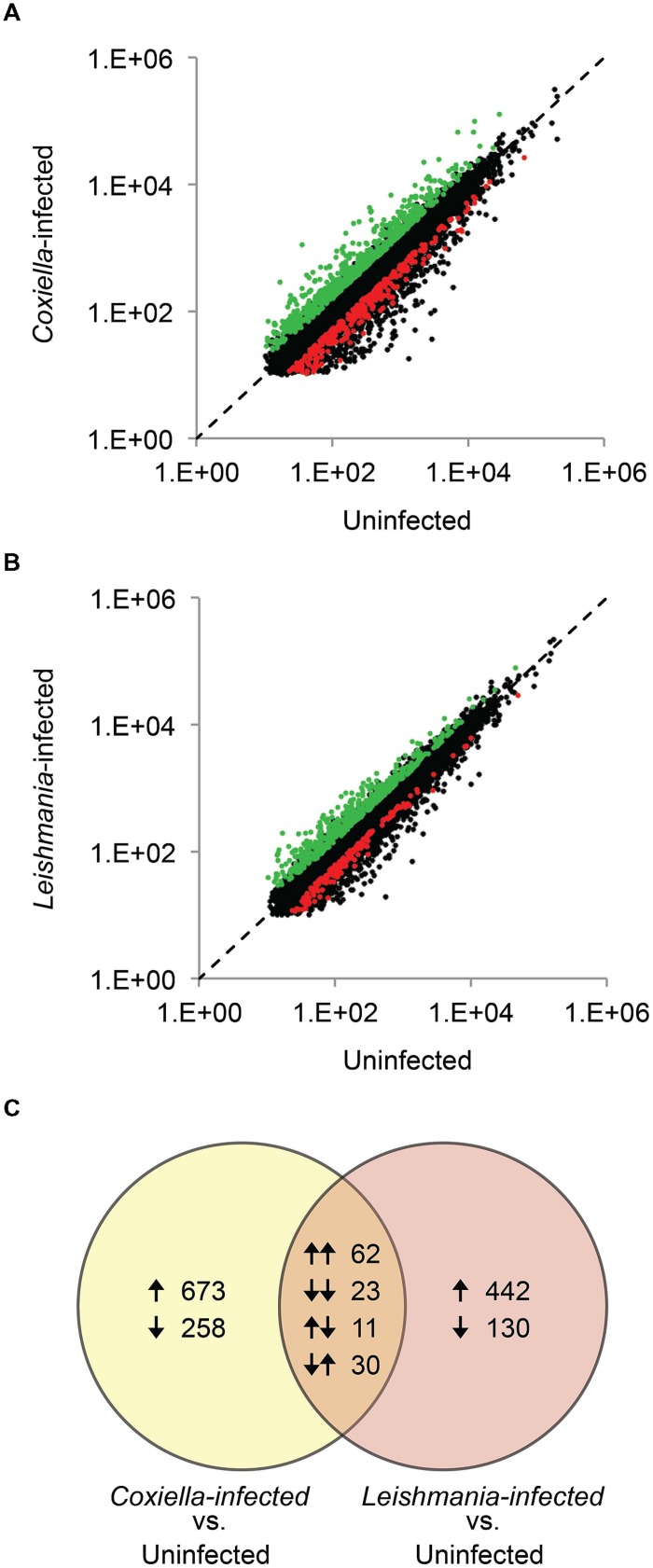
**Identification of differentially expressed genes.** Gene expression in **(A)** *Coxiella burnetii*-infected and **(B)** *Leishmania mexicana*-infected THP-1 cells in comparison to uninfected THP-1 cells are shown. Differentially expressed genes are highlighted in red and green. **(C)** Comparison of genes differentially expressed in *C. burnetii*-infected and *L. mexicana*-infected cells. Arrows indicate up-regulation or down-regulation of genes.

Human monocyte/macrophage cell line THP-1 was used to evaluate host responses against *C. burnetii* and *L. mexicana*. Previous studies have investigated host responses during early stages (6–72 hpi) of infections by *C. burnetii* and by various *Leishmania* species (Ren et al., 2003; Mahapatra et al., 2010; De Muylder et al., 2011; Rabhi et al., 2012, 2013); however, because the transformation from the infective form (SCV and promastigote, respectively) to the replicative form (LCV and amastigote, respectively) occur at differing rates in the two pathogens, we analyzed a later point during infection (5 days pi) when both pathogens have generated large PVs that fill most of the host cell volume. When compared to uninfected THP-1 cells, 1057 genes (746 up-regulated and 311 down-regulated) were differentially expressed in *C. burnetii* infected THP-1 cells, whereas 698 genes (534 up-regulated and 164 down-regulated) were differentially expressed in *L. mexicana* infected cells (**Figure 1**, **Supplementary Tables S1** and **S2**). Interestingly, the sets of genes affected by the two pathogens are very different. Of the 1755 total genes identified in this study, only 126 genes (~7%) are differentially expressed under both conditions, and no metabolic pathways were significantly enriched within this common set of genes (**Figure 1**, **Supplementary Table S3**). A previous study that compared THP-1 cell response to infections by *Coxiella* and *Chlamydia trachomatis* (an intracellular bacterium), reported an overlap of ~25% of genes between the two infections (Ren et al., 2003). The low overlap between the host responses to *Coxiella* and *Leishmania*, and the higher magnitude of host response to *C. burnetii* than that to *L. mexicana* possibly reflects the more distant evolutionary relationship between the bacteria and the eukaryotic parasite compared to the two bacterial pathogens previously studied. Apoptosis and host cell immune response pathways were the most significantly enriched KEGG pathways in *Coxiella* infected cells (**Table 1**), as observed in previous microarray-based studies (Ren et al., 2003; Mahapatra et al., 2010). Repression of host cell death by *Coxiella* has been reported previously (Lührmann and Roy, 2007; Voth et al., 2007), and is thought to promote intracellular growth of *Coxiella* within large PVs; conversely, induction of Toll-like Receptor signaling pathways and production of cytokines and chemokines participate in the host response to *Coxiella* infection.

**Table 1:** KEGG pathways enriched in *Coxiella*-infected and *Leishmania*-infected THP-1 cells.

Sample	KEGG term	Description	Genes	Fold enrichment	*P*-value
*Coxiella*-infected	hsa04210	Apoptosis	BID, IRAK2, TNF, XIAP, RELA, TP53, NFKBIA, ENDOD1, NFKB1, BIRC3, TNFRSF10A, CASP10, PRKAR2B, IRAK3, TNFRSF10B, PPP3CC, IL1B, PIK3R5, PIK3R3, IL1A	3.51	2.1E-06
	hsa04621	NOD-like receptor signaling pathway	CXCL1, TNF, XIAP, IL8, RELA, CXCL2, NFKBIA, NFKB1, BIRC3, NOD2, RIPK2, IL1B, TNFAIP3	3.20	5.4E-04
	hsa04060	Cytokine–cytokine receptor interaction	CXCL1, TNFRSF21, CCL3, TNF, CXCL5, CXCL3, CXCL2, TNFSF15, CXCL6, IL7R, CCL4, TGFB2, LIF, CCL22, IL23A, CCL20, CCL3L1, IL4R, TNFRSF18, IL15RA, IL1B, IL1A, BMP2, IL8, CD40, IL11RA, TNFRSF10A, INHBA, ACVR2B, TNFRSF10B, VEGFA	1.81	1.6E-03
	hsa04062	Chemokine signaling pathway	CXCL1, ADCY4, CCL3, LYN, CXCL5, IL8, HCK, CXCL3, RELA, CXCL2, NFKBIA, ADRBK2, NFKB1, CXCL6, CCL4, CCL22, CCL20, CCL3L1, GNG10, SOS2, PIK3R5, GNB4, PIK3R3, GNG7	1.96	2.2E-03
	hsa05222	Small cell lung cancer	E2F1, TRAF1, XIAP, PTGS2, RELA, TP53, ITGA2, NFKBIA, NFKB1, BIRC3, LAMB3, PIK3R5, PIK3R3, TRAF3	2.55	2.8E-03
	hsa05200	Pathways in cancer	TRAF1, E2F1, BID, PTGS2, XIAP, STAT5A, MITF, NFKBIA, NFKB1, NFKB2, TCF7L2, MMP1, TGFB2, LAMB3, SOS2, PIK3R5, CCNA1, PIK3R3, FGF2, TRAF3, BMP2, IL8, VHL, RELA, TP53, ITGA2, BIRC5, BIRC3, FZD4, DAPK3, CTNNA3, RAD51, SMO, ETS1, VEGFA	1.63	4.2E-03
	hsa04620	Toll-like receptor signaling pathway	CCL3, TNF, IL8, RELA, NFKBIA, NFKB1, CD40, CCL4, CD86, MAP3K8, IL1B, PIK3R5, PIK3R3, CD14, TRAF3	2.27	5.5E-03
*Leishmania*-infected	hsa00230	Purine metabolism	ADCY4, ADSSL1, ADCY8, POLA1, PDE4C, PDE6G, POLE4, PDE2A, ADCY9, RRM2, PKLR, GUCY1A2, ADCY10, PRPS1	2.40	5.1E-03

In *Leishmania*-infected cells, purine metabolism was the only KEGG pathway that was significantly perturbed (**Table 1**). *Leishmania* is dependent on host for its purine supply (McConville et al., 2007), and three genes (ADSSL1, RRM2, PRPS1) involved in purine biosynthesis or salvage pathways were significantly overexpressed in infected THP-1 cells. Intriguingly, a majority of “purine metabolism” genes listed in **Table 1** regulate the levels of intracellular second messengers cAMP and cGMP. Adenylate cyclases (ADCY4, ADCY8, ADCY9, ADCY10) catalyze the formation of cAMP from ATP; guanylate cyclase (GUCY1A2) catalyzes the conversion of GTP to cGMP; phosphodiesterases (PDE4C, PDE6G, PDE2A) catalyze the hydrolysis of cAMP and/or cGMP. Previous studies have shown that *Leishmania* resists host antimicrobial activities by modulating several host signaling pathways, including Ca^2+^- and PKC-dependent pathways, JAK-STAT pathways, and MAP kinases (Olivier et al., 2005). Similarly, *Leishmania* could be subverting the host’s cAMP and cGMP signaling pathways in order to suppress immune responses and to promote its intracellular growth.

**FIGURE 2:**
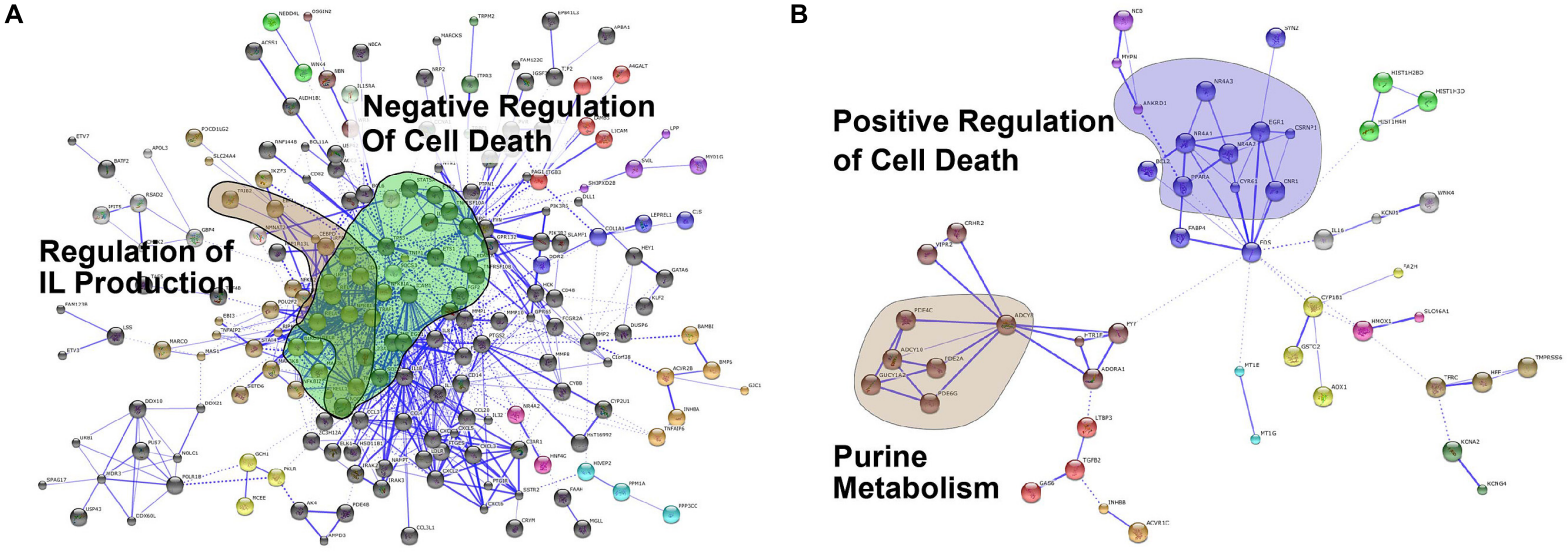
**Protein-protein interaction analysis.** Protein-protein interaction networks of up-regulated genes in **(A)** *C. burnetii*-infected and **(B)** *L. mexicana*-infected THP1 cells visualized in STRING. Colors based on Markov Clustering with an inflation factor of 2. Highlighted clusters are labeled with their GO or KEGG categories.

A protein–protein interaction network analysis using the STRING database (Franceschini et al., 2013) confirmed that *Coxiella* infection induced the expression of genes involved in negative regulation of cell death (**Figure 2A**). In contrast, this analysis identified that genes involved in positive regulation of cell death were upregulated in *Leishmania*-infected cells (**Figure 2B**). We confirmed this trend by analyzing the expression of a subset of cell death-related genes using qPCR (Additional File **Supplementary Figure S1**). The induction of host cell death during later stages of infection probably aids in the cell-to-cell transfer of *Leishmania* amastigotes within membrane blebs, as shown recently (Real et al., 2014).

### Differential Expression of mRNA Isoforms in Infected and Uninfected Cells

In human cells, alternate splicing of pre-mRNA can give rise to several isoforms of the mature mRNA, and proteins derived from them may have distinct cellular roles (Lareau et al., 2004). In addition to expanding the proteome, cells utilize alternate splicing as a regulatory tool. For example, a short splice variant of human tryptophan-tRNA synthase, but not the full length protein, regulates angiogenesis (Wakasugi et al., 2002). Isoform generation may also have a role in host cell response against infections. Different isoforms of p53 (encoded by TP53 gene) are involved in host defense against both bacterial (*Helicobacter pylori*) and viral (Influenza and Simian virus 40) infections (Terrier et al., 2013). Similarly, Hepatitis C virus activates the immunologic isoform of nitric oxide synthase (NOS) gene, which induces NO production (Machida et al., 2004).

**FIGURE 3:**
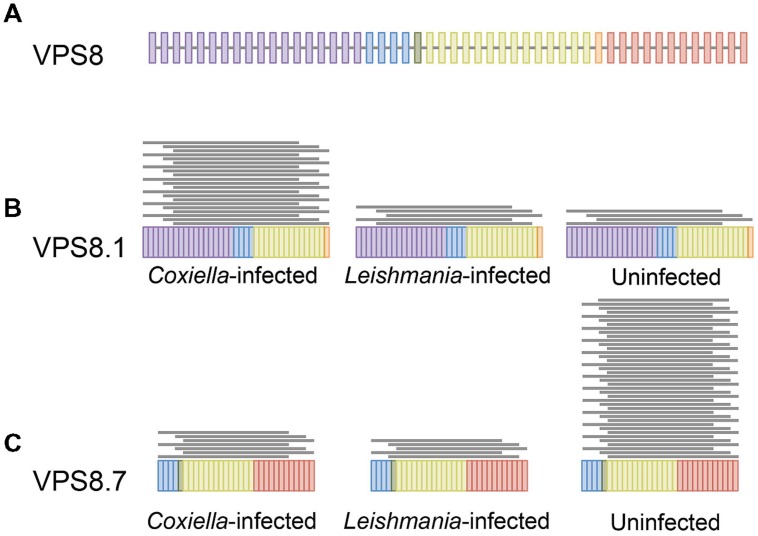
**Differential expression of mRNA isoforms.** Differential isoform expression of VPS8 in *C. burnetii* infected, *L. mexicana* infected, and uninfected THP1 cells are shown as a representation of isoform analysis. **(A)** The full-length VPS8 gene is depicted with colored bars representing exons. Isoforms 1 **(B)** and 7 **(C)** of VPS8 that have significantly different expression in the three samples are shown. Each gray line above an isoform represents 10 mapped reads.

Transcriptome analysis (RNA-seq) is a powerful approach to identify differential isoform expression under different conditions at a genome-wide scale (Eswaran et al., 2013; Lo et al., 2014). We used RNA-seq to investigate whether infection by either *Coxiella* or *Leishmania* induced differential expression of human gene isoforms. We identified 689 isoforms from 626 genes that were differentially expressed in *C. burnetii*-infected cells, and 651 isoforms from 569 genes in *Leishmania*-infected cells, when compared to uninfected THP-1 cells (**Figure 3**, **Supplementary Tables S4** and **S5**). As observed for full-length mRNAs, there was minimal overlap between the sets of genes with differential expression of isoforms under each infection condition (only 105 common genes). Additionally, no KEGG pathways were significantly enriched in either gene set, indicating that differential isoform expression is a cell-wide phenomenon. Cumulatively, our data revealed that in addition to differences that are apparent at the gene level, the mostly unexplored realm of isoform variation could contribute to host responses to infections.

### *Coxiella* and *Leishmania* Infections Perturb the Expression of Apoptosis-Related miRNAs

Expression of various protein-coding genes in humans is regulated by miRNAs. These small non-coding RNAs regulate the expression of target genes by base-pairing with mRNAs, thereby either blocking translation or causing target degradation or destabilization (Fabian et al., 2010). They are involved in many, if not all, biological processes, including metabolic pathways, cell proliferation, and apoptosis. Recently, miRNAs have been shown to be an important part of host cell response to viral, bacterial, and parasitic infections (Lagos et al., 2010; Schnitger et al., 2011; Schulte et al., 2011). In addition, some viruses, including Herpes viruses and Hepatitis C virus, have the ability to interfere with the host miRNA network to promote viral growth (Jopling et al., 2005; Cullen, 2011). Recent studies also showed that eukaryotic intracellular pathogens such as *Cryptosporidium parvum* and *T. gondii* promote intracellular replication by altering host cell miRNA networks (Hakimi and Ménard, 2010; Zeiner et al., 2010). To identify miRNAs that are potentially perturbed by *C. burnetii* or *L. mexicana* infections, we sequenced and enumerated miRNAs expressed by uninfected, *Coxiella*-infected, and *Leishmania*-infected THP-1 cells. We identified 257 miRNAs that were expressed in THP-1 cells (**Supplementary Table S6**), which includes 50 of the 64 miRNAs reported by a recent study that examined miRNAs expressed in human macrophages in response to *Leishmania major* infection (Lemaire et al., 2013). Among the 257 miRNAs, seven were upregulated and one was down regulated in *Coxiella*-infected cells, and three were upregulated and two were down regulated in *Leishmania*-infected cells (**Table 2**). Intriguingly, several of the differentially expressed miRNAs have been shown in previous studies to regulate host cell death: miR-145 modulates the expression of KLF4 (Davis-Dusenbery et al., 2011), a transcription factor for TP53, which regulates apoptosis (Rowland et al., 2005); miR-15b and miR-29b are known to be pro-apoptotic in leukemia cells (Cimmino et al., 2005; Garzon et al., 2009); miR-148a promotes apoptosis by targeting BCL2 in colorectal cancer cells (Zhang et al., 2011); miR-181d also targets BCL-2 and promotes apoptosis in glioma cells (Wang et al., 2012). These results complement gene expression data (**Figure 2**), and indicate that miRNAs may have important roles in inhibiting host cell death during *Coxiella* infection, and promoting host cell death during *Leishmania* infection.

**Table 2:** MicroRNAs (miRNAs) perturbed by *Coxiella* and *Leishmania* infections.

Sample	miRNA	Fold change (log2)	*P-*value	Regulation	Process
*Coxiella*-infected	mir-148a-3p	-0.58	0.024	Down	Pro-apoptotic^a^
	mir-181d-5p	0.78	<0.001	Up	Anti-apoptotic^b^
	mir-193a-5p	0.81	<0.001	Up	Pro-apoptotic^c^
	mir-362-5p	0.89	0.015	Up	Anti-apoptotic^d^
	mir-361-5p	0.95	0.004	Up	Anti-apoptotic^e^
	mir-194-2-5p	1.05	0.024	Up	Anti-apoptotic^f^
	mir-28-3p	1.12	0.024	Up	Neither^g^
	mir-28-5p	1.35	<0.001	Up	Pro-apoptotic^g^
*Leishmania*-infected	mir-145-5p	-1.00	0.002	Down	Pro-apoptotic^h^
	mir-221-5p	-0.62	<0.001	Down	Anti-apoptotic^i^
	mir-15b-5p	0.56	0.035	Up	Pro-apoptotic^j^
	mir-29b-1-3p	1.09	0.002	Up	Pro-apoptotic^k^
	mir-29b-2-3p	1.19	<0.001	Up	Pro-apoptotic^k^

*^a^Zhang et al. (2011), ^b^Wang et al. (2010), ^c^Nakano et al. (2013), ^d^Xia et al. (2014), ^e^Wu et al. (2013), ^f^Zhang et al. (2014), ^g^Almeida et al. (2012), ^h^Davis-Dusenbery et al. (2011), ^i^le Sage et al. (2007), ^j^Cimmino et al. (2005), ^k^Garzon et al. (2009).*

## Conclusion

The genome-wide gene, mRNA-isoform, and miRNA expression patterns were distinct between macrophages infected with either *C. burnetii* or *L. mexicana*, indicating that even though both pathogens have converged on a similar intracellular niche, they utilize distinct programs to generate and maintain their respective PVs.

## Author Contributions

RR, EC, and SL designed the study. RV, FK, and EC carried out the experiments. JM and RR analyzed the data and drafted the manuscript. All authors read and approved the final manuscript.

## Conflict of Interest Statement

The authors declare that the research was conducted in the absence of any commercial or financial relationships that could be construed as a potential conflict of interest.

## Supplementary Material

FIGURE S1 **Validation by qPCR of expression levels estimated by RNA-seq. Fold difference values calculated by qPCR correlated well with fold difference values calculated by RNA-seq**.

TABLE S1 **Differentially expressed genes in *Coxiella*-infected vs. uninfected THP-1 cells**.

TABLE S2 **Differentially expressed genes in *Leishmania*-infected vs. uninfected THP-1 cells**.

TABLE S3 **Differentially expressed genes that overlap between *Coxiella*-infected vs. uninfected and *Leishmania*-infected vs. uninfected THP-1 cells**.

TABLE S4 **Differentially expressed mRNA isoforms in *Coxiella*-infected vs. uninfected THP-1 cells**.

TABLE S5 **Differentially expressed mRNA isoforms in *Leishmania*-infected vs. uninfected THP-1 cells**.

TABLE S6 **All miRNAs detected in this study**.
