# Supplementary figures and images for: Coxiella burnetii and Leishmania mexicana residing within similar parasitophorous vacuoles elicit disparate host responses
Source: Front Microbiol. 2015 Aug 7;6:794. doi: 10.3389/fmicb.2015.00794 (PMC4528172; doi:10.3389/fmicb.2015.00794)

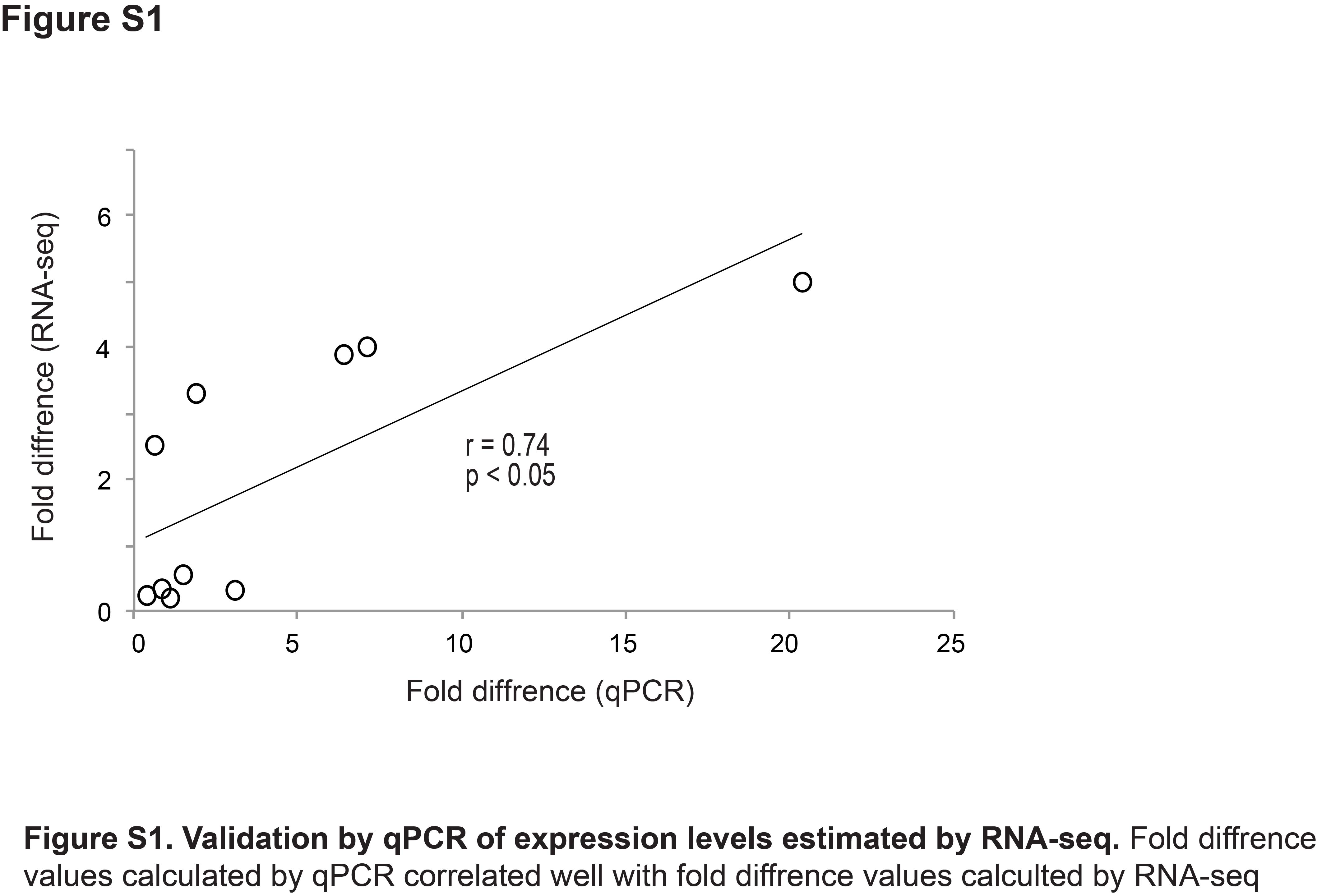

Supplement: FIGURE S1 — Validation by qPCR of expression levels estimated by RNA-seq. Fold difference values calculated by qPCR correlated well with fold difference values calculated by RNA-seq. [file Image_1.JPEG]
